# Clinical and Inflammatory Outcomes Following Multimodal Anesthesia and On-Table Extubation in Elderly Patients Undergoing Elective Cardiac Surgery with (Partial) Sternotomy: A Secondary Analysis of RERACS Study

**DOI:** 10.3390/jcm15218451

**Published:** 2026-10-30

**Authors:** Hannah Maria Conrads, Adelina Werner, Maria Tafelmeier, Bernhard Flörchinger, Michael Arzt, Diane Bitzinger

**Affiliations:** 1 Department of Anesthesiology, University Hospital Regensburg, Franz-Josef-Strauss-Allee 11, 93053 Regensburg, Germany; adelina.werner@stud.uni-regensburg.de (A.W.); diane.bitzinger@ukr.de (D.B.); 2 Department of Internal Medicine II, University Hospital Regensburg, Franz-Josef-Strauss-Allee 11, 93053 Regensburg, Germany; maria.tafelmeier@ukr.de (M.T.); michael.arzt@ukr.de (M.A.); 3 Department of Cardiothoracic Surgery, University Hospital Regensburg, Franz-Josef-Strauss-Allee 11, 93053 Regensburg, Germany; bernhard.floerchinger@ukr.de

**Keywords:** Enhanced Recovery After Cardiac Surgery, on-table extubation, demographic change, IL-6, patient-reported outcome measure, perioperative care, resource-aware, multimodal anesthesia, sternotomy

## Abstract

**Background**: Enhanced Recovery After Cardiac Surgery (ERACS) is a multimodal approach aiming for optimized postoperative outcomes. Regarding demographic change and the epidemiology of heart diseases, ERACS concepts with on-table extubation (OTE) might be a resource-aware solution needed to ensure adequate perioperative surgical care to an aging society. We investigated clinical outcomes and inflammatory effects of ERACS with multimodal anesthesia and OTE in patients >65 years undergoing elective cardiac surgery. **Methods**: This study is a secondary analysis of the RERACS study (Regensburg Enhanced Recovery After Cardiac Surgery). Sixty-four elderly RERACS patients were compared with a retrospective control group (age >65 years, *n* = 67) undergoing elective cardiac surgery via (partial) sternotomy. The RERACS concept consisted of shortened fasting periods, breathing training, opioid-sparing multimodal analgesia, OTE, patient-reported outcome measures and additional exploratory evaluation of Interleukin-6 (IL-6) and TNF-alpha in this secondary analysis. The control group received standard care with extubation in the intensive care unit (ICU). **Results**: Unadjusted baseline analysis showed no significant differences between the two groups. Length of stay in ICU (ICU-LOS) and postoperative catecholamine weaning were significantly shorter in the RERACS group. RERACS patients showed significantly lower C-reactive-protein levels 24 h after surgery than control patients. In the RERACS group, postoperative low interleukin-6 levels were associated with postoperative complications like delirium or infection, but not with atrial fibrillation. Patient-reported outcome measures in the RERACS group showed high patient satisfaction, good pain compensation and low levels of postoperative nausea and vomiting but remarkable levels of sore throat/hoarseness, despite shorter ventilation time. **Conclusions**: Data support that ERACS with OTE is feasible in selected elderly patients undergoing elective cardiac surgery via (partial) sternotomy. No apparent increase in required postoperative respiratory support occurred compared to patients with conservative, prolonged mechanical ventilation. The RERACS program with multimodal anesthesia and OTE was associated with shorter ICU-LOS, earlier weaning from vasoactive support, and high patient satisfaction. Further studies are needed to evaluate immunomodulatory effects of ERACS measures and investigate the role of IL-6 levels as a marker for immunoparalysis in elderly cardiosurgical patients.

## 1. Introduction

While global life expectancy has already increased by 6.2 years since 1990 [1], forecasts reveal that the average life expectancy will continue increasing [1] by another 4.8 years from 2022 to 2050 [2]. Considering the WHO report “The Global Burden of Disease: Update 2021”, ischemic heart disease will still be the leading cause of disease burden worldwide in 2050 [2]. Regarding demographic change and the correlation between common heart diseases and higher age, individualized, resource-aware perioperative concepts are needed to ensure adequate cardiac surgical care to an aging society [1,3,4]. Given the limited healthcare resources due to staffing shortages, a more efficient utilization of intensive care unit (ICU) facilities is essential to address the growing demands and to minimize treatment delays.

Enhanced Recovery After Cardiac Surgery (ERACS) is a multimodal and interdisciplinary approach [5] for optimizing perioperative management and patient outcomes [3]. The 2019 ERAS Cardiac Society update by Engelmann et al. [5] and the 2024 joint consensus statement [6] codified more than 30 evidence-based elements aimed at accelerating recovery, but the evidence for timing of postoperative extubation is pending and has led to an ongoing discussion in the professional community [7,8,9]. By minimizing the surgical [6] and anesthesiologic stress, ERACS concepts might have positive effects on the patients’ perioperative immunocompetence and homeostasis. However, there are mainly studies pointing out the benefits of ERACS concepts with on-table extubation (OTE) in younger patients or patients undergoing minimal invasive cardiac surgery, which show improved postoperative surgical outcomes [6]. Furthermore, outcome evaluation in ERACS so far mainly focuses on outcomes like length of stay (LOS) and postoperative complications. But postoperative recovery is a complex process with multiple relevant domains, including physiological, nociceptive, mental health, cognitive, sleep and mobility. So, there are increasing calls for the use of patient-reported outcome measures (PROMs) to improve the recovery process and to adjust perioperative patient pathways. Especially elderly cardiac surgical patients may also benefit from developing patient-centered, multidisciplinary ERACS protocols including PROMs. Hence, this secondary analysis of the Regensburg Enhanced Recovery After Cardiac Surgery (RERACS) study (DRKS00031402) [10] was carried out to evaluate whether an ERACS protocol incorporating multimodal anesthesia and OTE improves clinical outcomes and attenuates postoperative inflammation in elderly patients undergoing elective cardiac surgery with (partial) sternotomy.

## 2. Materials and Methods

The current study is a secondary analysis of the RERACS study (registered at German Clinical Trials Register, DRKS00031402). A total of 146 patients were initially included in the RERACS group from April 2023 to December 2024. While the primary analysis (RERACS population analyzed from April 2023 to 3 September 2024) focused on patients aged 18 years to 75 years, in this secondary analysis, we analyzed an elderly patient population aged over 65 years. The cohort overlap with the primary analysis in the RERACS group was 49.6% (*n* = 59). The directional overlap of the RERACS patients in the primary analysis (*n* = 114) included in the secondary analysis was 51.8% (*n* = 59) whereas 92.2% (*n* = 59) of the secondary analyzed RERACS patients (*n* = 64) had been included in the primary analysis. For the control group, the cohort overlap with the primary analysis was 25.7% (*n* = 38). The directional overlap of control patients in the primary analysis (*n* = 119) included in the secondary analysis was 31.9% (*n* = 38), whereas 56.7% (*n* = 38) of the secondary analyzed control patients (*n* = 67) had been included in the primary analysis. In this secondary analysis, all 64 RERACS patients aged >65 years were compared to a retrospective control group aged >65 years (*n* = 67) included from January 2022 to March 2023. The control group analyzed in the primary analysis was included from July 2022 to March 2023. The RERACS patients received a multimodal package of perioperative measures, while the conventionally treated control group received standard therapy. Both groups underwent elective cardiac surgery such as aortic valve replacement (AVR) and coronary artery bypass surgery (CABS) by (partial) sternotomy. Consistent with the primary analysis, further inclusion criteria were (1) patients’ informed consent in the RERACS group, (2) body mass index (BMI) between 15 and 35 kg/m^2^, (3) left ventricular ejection fraction >30%, (4) no relevant comorbidities such as chronic obstructive pulmonary disease, cirrhosis of the liver, New York Heart Association class IV or chronic renal failure requiring dialysis. In case of (1) emergency interventions, (2) re-surgery, (3) surgery with a higher risk of postoperative secondary bleeding, (4) intraoperative hypothermia lower than 32 degrees, (5) preoperative necessity of catecholamine therapy or other cardiac assist systems, (6) surgery during acute endocarditis, (7) relevant mental or neurological pathologies, (8) postoperative cardiopulmonary instabilities or (9) severe hearing or visual impairment without visual or hearing aids, patients were excluded from the study. Postoperative cardiopulmonary instability was defined as hemodynamic or respiratory complications appearing during/after weaning from cardiopulmonary bypass (CPB) such as hemodynamically significant arrhythmias or persistent hypotension requiring escalation of the standard catecholamine regime. A detailed patient-flow diagram was reported previously by Werner et al. showing failure in timely extubation of 19 RERACS patients (of which geriatric: *n* = 10) due to high risk of postoperative bleeding and cardiopulmonary instability [10].

### 2.1. Parameters Recorded and Their Definition

The primary endpoint was postoperative LOS. Length of stay in the ICU (ICU-LOS) and total duration of postoperative stay in hospital (hospital LOS) were evaluated using SAP (SAP Deutschland SE & Co. KG, Walldorf, Germany).

Secondary measurements were length of postoperative vasoactive drug support, duration of mechanical ventilation, complication rate, and patient satisfaction on the second postoperative day. The duration of catecholamine therapy was defined from the end of surgery to the end of any inotrope or vasopressor medication given in the ICU and intermediate care unit (IMC). Total invasive ventilation time was defined as postoperative mechanical ventilation in the ICU until extubation. Non-invasive ventilation (NIV) time was examined using the patient data management systems (PDMS) during treatment in the ICU and IMC.

As complications, we recorded retrospectively (1) postoperative nausea and vomiting (PONV), (2) (subsyndromal) delirium, (3) redo-surgery, (4) unplanned re-intubation, (5) new-onset atrial fibrillation (AFib), and (6) postoperative infection. PONV was recorded if a total dose of 8 mg ondansetron was exceeded or other antiemetic drugs were administered. To detect postoperative delirium, the Intensive Care Unit Delirium Screening Checklist (ICDSC) [11] was routinely calculated at least once per nursing shift. Delirium was divided into no delirium (ICDSC = 0), subsyndromal delirium defined as ICDSC 1–3, and syndromic delirium defined as ICDSC ≥ 4. Redo-surgery included pacemaker implantation as well as surgery due to secondary bleeding. Postoperative new-onset AFib was recorded if medical or electric measures such as administration of amiodarone or cardioversion were taken. Postoperative infection was defined if a newly initiated administration of antibiotics was necessary during the postoperative course.

Additionally, exploratory evaluation of inflammatory markers was part of this secondary analysis. Preoperative and postoperative C-reactive protein (CRP) and N-terminal pro-brain natriuretic peptide (NTproBNP) values were part of the perioperative routine blood screening and were taken on the day before and after surgery. CRP was analyzed from heparinized plasma using immunoturbidimetry. NTproBNP was analyzed from heparinized plasma using electrochemiluminescence immunoassay. For further inflammatory characterization, Interleukin 6 (IL-6) and Tumor-necrosis-factor α (TNF-α) were evaluated in the RERACS group from blood samples drawn at the induction of anesthesia and at the end of surgery, as well as 24 h postoperatively. For IL-6 and TNF-α analysis, serum blood samples were drawn and immediately sent to the central laboratory of the University Hospital of Regensburg without refrigeration. Analysis was performed using electrochemiluminescence immunoassay within 1 h after receipt. Lower limits of detection were 0.6 mg/L for CRP, 50 pg/mL for NTproBNP, 2.5 pg/mL for IL-6 and 4 pg/mL for TNF-α. After analysis, the samples were discarded.

To assess patient satisfaction after surgery and PROMs, a standardized questionnaire [12] was used on the second postoperative day. The questionnaire consisted of 12 questions concerning the common patient complaints after general anesthesia such as PONV, shivering, pain, sore throat, postoperative cognitive dysfunction and intraoperative awareness as well as areas of patient satisfaction (e.g., extent of the preoperative consultation, pain management). Evaluation of PROMs was first introduced within the implementation of the RERACS concept.

Although the current study is a secondary analysis of the RERACS study, no post hoc analyses were performed. All endpoints and exploratory measurements were specified in the study protocol from the outset.

### 2.2. Perioperative Treatment Concept of the RERACS Group

As reported previously by Werner et al., the RERACS concept consisted of three perioperative pillars with multimodal analgesia and OTE [10]. Preoperatively, the RERACS patients received an education program aiming, e.g., for optimization of the patients’ cardiorespiratory function [3] by using the COACH-breathing trainer. Another main aspect was shortening the preoperative fasting period. Patients were encouraged to eat until 12 pm the day before surgery and to drink clear (sweetened) liquids 2 h before surgery. The use of sedative medication was avoided preoperatively. The first blood sample to evaluate the preoperative levels of IL-6 and TNF-α was taken before induction of anesthesia. Intraoperative anesthesiologic management consisted of opioid-sparing multimodal analgesia (sufentanil (Piramal Ciritcal Care Deutschland GmbH, Hallbergmoos, Germany) boluses 1 µg/kg for the entire surgery, remifentanil (GlaxoSmithKline GmbH & Co. KG, Munich, Germany) infusion 0.5 µg·kg^−1^·min^−1^, dexmedetomidine (Pharmacy of University Hospital Regensburg, Regensburg, Germany) 40 µg/h, 1 g metamizole (Zentiva Pharma GmbH, Frankfurt am Main, Germany), low-dose ketamine (PANPHARMA GmbH, Trittau, Germany) boluses (0.2 mg·kg^−1^·h^−1^) including parasternal block (20 mL ropivacaine 7.5 mg/mL, Sintetica GmbH, Münster, Germany). PONV prophylaxis (4–8 mg dexamethasone, hameln pharma GmbH, Hameln, Germany; 4 mg ondansetron, Fresenius Kabi Deutschland GmbH, Bad Homburg, Germany) and temperature management were performed as reported previously [10]. Following weaning from CPB, the team made a joint decision regarding the extubation strategy in RERACS patients—either proceeding with OTE or transporting the patient intubated to the ICU. The decision for OTE was made on a case-by-case basis by the attending anesthesiologist in agreement with the surgical team after separation from CPB. OTE was not solely determined by the type of surgery, CPB duration, cross-clamp time, or comorbidities but rather based on hemodynamic stability, bleeding complications and respiratory and thermal stability. OTE was never performed if anyone on the team was concerned about the patient’s safety. Postoperatively, the extubated patients were moved to the ICU, where inspiratory muscle training using COACH was recommenced as soon as possible. If necessary, non-invasive ventilation (NIV) (mask and/or High Flow) was introduced. During the stay in the ICU, early mobilization within 6 h postoperatively and shortened postoperative fasting periods commencing oral fluid or ice water intake within 6 h after surgery was aimed for. The ICDSC was routinely calculated at least once per nursing shift in both the RERACS group and the control group. Furthermore, we aimed for early removal of drains and catheters as needed. To assess the patient’s satisfaction, a standardized questionnaire was used on the second postoperative day. Twenty-four hours after surgery, a third blood sample was taken to evaluate the postoperative course of IL-6 and TNF-α. Perioperative laboratory parameters were evaluated as shown in Table 1.

The control group received perioperative standard care, using opioid-based intraoperative management with sufentanil infusion (1 µg·kg^−1^·h^−1^). The definition of cardiopulmonary instability was applied identically to both the RERACS group and the historical control cohort. Control patients requiring an escalation of the standard catecholamine regimen at the end of surgery were excluded from the control group. Patients in the control group were sedated with propofol (3 mg·kg^−1^·h^−1^) during transfer and until extubation in the ICU.

### 2.3. Statistical Analysis

Data analysis was based on electronic patient files including an electronic patient data management system and medical records (MetaVision v6 (iMDsoft, Düsseldorf, Germany) and SAP).

Statistical analyses were performed using IBM SPSS Statistics v29 (SPSS Inc.: Chicago, IL, USA). For the descriptive statistics, mean ± standard deviation (SD) was used for presenting symmetric variables. Mean ± standard deviation (SD) and median and interquartile range (IQR) were used for presenting skewed variables. In the exploratory data analysis, the differences between the two treatment groups (control vs. RERACS) were determined using two-sample *t*-test for symmetric variables. For skewed data, the nonparametric rank-based Mann–Whitney U test was used to determine differences between the RERACS group and control group. To compare the frequencies in characteristics between the two groups, Pearson’s chi-squared test was used. Standardized mean differences in characteristics of both study groups were calculated using Cohen’s d and Cramer’s V. Inflammatory analysis is novel in this current secondary analysis. Therefore, to compare inflammatory markers between the RERACS group and control group in this secondary analysis, biserial correlation was additionally used with 95% confidence intervals (CIs). Spearman rank correlation was of novel use for analysis of association between inflammatory markers and postoperative complications in the RERACS group (ranges from −1 to +1). Receiver operating characteristic (ROC) curve analysis was conducted to calculate the diagnostic accuracy of IL-6 values using area under the curve (AUC). To identify exploratory clinical cut-off values of IL-6, the Youden Index was calculated. The Jaccard Index was used to calculate the degree of overlap in the study groups of the original analysis and secondary analysis. A *p*-value < 0.05 was considered statistically significant.

### 2.4. Ethical Approval

The study protocol and the consent form were approved by the institutional ethics committee of the University Hospital of Regensburg on 1 March 2023 (ethics vote: EA 23-3270-101). For the RERACS group, informed consent was obtained, whereas for the retrospective control group, this was not deemed necessary, as no identifying data were collected. The study was (retrospectively) registered at German Clinical Trials Register on 20 October 2023 (ID: DRKS00031402; URL: https://drks.de/search/en/trial/DRKS00031402/details, accessed on 19 October 2026).

## 3. Results

### 3.1. Baseline Data

In this secondary analysis, 64 RERACS patients aged >65 years were compared to a retrospective control group aged >65 years (*n* = 67) included from January 2022 to March 2023.

Table 2 demonstrates a general overview of the distribution of various preoperative and intraoperative characteristics of the RERACS group and the control group. Both groups underwent elective cardiac surgery. In the RERACS group, 22 (34.4%) patients underwent AVR surgery, and 42 (65.6%) patients underwent CABS with no significant difference compared to the control group (AVR: 23 (34.3%) patients, CABS: 44 (65.7%) patients). Furthermore, unadjusted analysis showed no significant difference in terms of age, sex, BMI, preoperative ejection fraction, or comorbidities as well as type (partial sternotomy: 17.4% vs. 14.9%; *p* = 0.724) and duration of operation between the two groups. Calculating effect size showed mostly slight to moderate differences in characteristics of the two study groups. However, time until OR exit was significantly longer in the RERACS group than in the control group (20.84 ± 6.94 min vs. 15.36 ± 5.39 min; *p* < 0.001; d = 0.884).

### 3.2. Primary Results: Clinical Outcomes

Table 3 demonstrates postoperative LOS in ICU and hospital as primary end points of the current study. While hospital LOS (9.8 ± 4.4 days vs. 11.5 ± 7.7 days; *p* = 0.155) was comparable between the two groups, the ICU-LOS was significantly shorter in the RERACS group (44.5 ± 39.8 h vs. 63.2 ± 48.4 h; *p* = 0.003). Furthermore, postoperative catecholamine weaning was significantly faster in the RERACS group than in the control group (29.51 ± 33.19 h vs. 50.62 ± 54.79 h; *p* = 0.008). While all control patients required postoperative circulatory support using inotropes and vasopressors, only 82.5% of the RERACS patients received catecholamines postoperatively (*p* < 0.001).

Aiming for OTE by design, mechanical ventilation time was significantly shorter in the RERACS group compared with the control group (16.42 ± 130.62 min vs. 855.63 ± 783.78 min; *p* < 0.001, Table 3). The mechanical ventilation time in the RERACS group was based on the mechanical ventilation of two RERACS patients after redo-surgery because of secondary bleeding in one patient and pacemaker implantation in the other patient. However, there were no significant differences regarding the non-invasive ventilation times and prevalences between the two groups. Unplanned reintubation due to respiratory insufficiency was necessary in one of the control patients but none of the RERACS patients (Table 3).

Postoperative oxycodone requirements (20.72 ± 17.75 mg vs. 19.39 ± 13.80 mg; *p* = 0.865) were not significantly different between the two groups. Moreover, the RERACS group showed a significantly lower incidence of postoperative infection (26.6% vs. 46.3%; *p* = 0.019) and PONV (4.8% vs. 35.8%; *p* < 0.001). The rates of unplanned redo-operations (3.1% vs. 9.0%) as well as (subsyndromal) delirium (48.4% vs. 59.7%) were lower in the RERACS group but reached no statistical significance.

### 3.3. Secondary Results: Patient-Reported Outcome Measures (PROMs) in RERACS Group

On the second postoperative day, a standardized questionnaire was used to assess postoperative patient satisfaction and PROMs in the RERACS group. The 12 questions targeted frequent general patient complaints after general anesthesia (Table 4) as well as duration of preoperative fasting periods regarding food and liquid intake. As shown in Table 4, most patients in the RERACS group were very satisfied with the postoperative pain management (92.3%). Patient care, friendliness of staff and preoperative patient information were also evaluated with high patient satisfaction. Only a few patients complained about postoperative shivering (1.9%) as well as nausea and vomiting (3.8%). Additionally, 7.7% of the patients reported pain outside the surgical site, mainly in the back or shoulder area. However, sore throat and hoarseness (completely 13.5%, somewhat 11.5%) were the main postoperative complaints patients reported after cardiac surgery in the RERACS group. Subjective perception of confusion and cognitive dysfunction (34.6%) was slightly lower than the objective detection of (subsyndromal) delirium via ICDSC classification (48.4%) in PDMS.

### 3.4. Exploratory Results: Inflammatory Outcomes

Preoperative and postoperative CRP and NTproBNP values were part of the perioperative routine blood screening and were taken on the day before and 24 h after surgery (Table 5). While there was no significant difference in expression of NTproBNP levels pre- and postoperatively, postoperative CRP levels were significantly higher in the control group than in the RERACS group (105.87 ± 60.13 mg/L vs. 64.73 ± 53.34 mg/L; *r* = −0.391 [95% CI −0.487, −0.285]; *p* < 0.001). Preoperatively, there was no significant difference in CRP levels between the two groups (*p* = 0.485).

For postoperative delirium, the receiver operating characteristic (ROC) curve analysis showed no statistically significant discriminatory power of IL-6 levels at induction of anesthesia and end of surgery (Table 6). However, 24 h postoperatively, lower exploratory IL-6 levels were associated with a higher probability of postoperative delirium (AUC = 0.716; 95% CI: 0.549–0.883; *p* = 0.025). For postoperative infection, the ROC analysis showed no statistically significant discriminatory power of exploratory IL-6 levels at induction of anesthesia and end of surgery (Table 6). However, 24 h postoperatively, lower exploratory IL-6 levels were associated with a higher probability of postoperative infection (AUC = 0.856; 95% CI: 0.715–0.996; *p* = 0.002). For postoperative new-onset AFib, the ROC analysis showed no statistically significant discriminatory power of exploratory IL-6 levels at any perioperative time (Table 6). Calculating the Youden Index yielded an exploratory cut-off value of 56.2 pg/mL (sensitivity: 50%, specificity: 88.2%, Youden Index = 0.382) for 24 h IL-6 levels in patients with postoperative delirium. For patients with postoperative infection, calculating the Youden Index yielded an exploratory cut-off value of 84.95 pg/mL (sensitivity: 100%, specificity: 58.8%, Youden Index = 0.588) for 24 h IL-6 levels.

**Figure 1:**
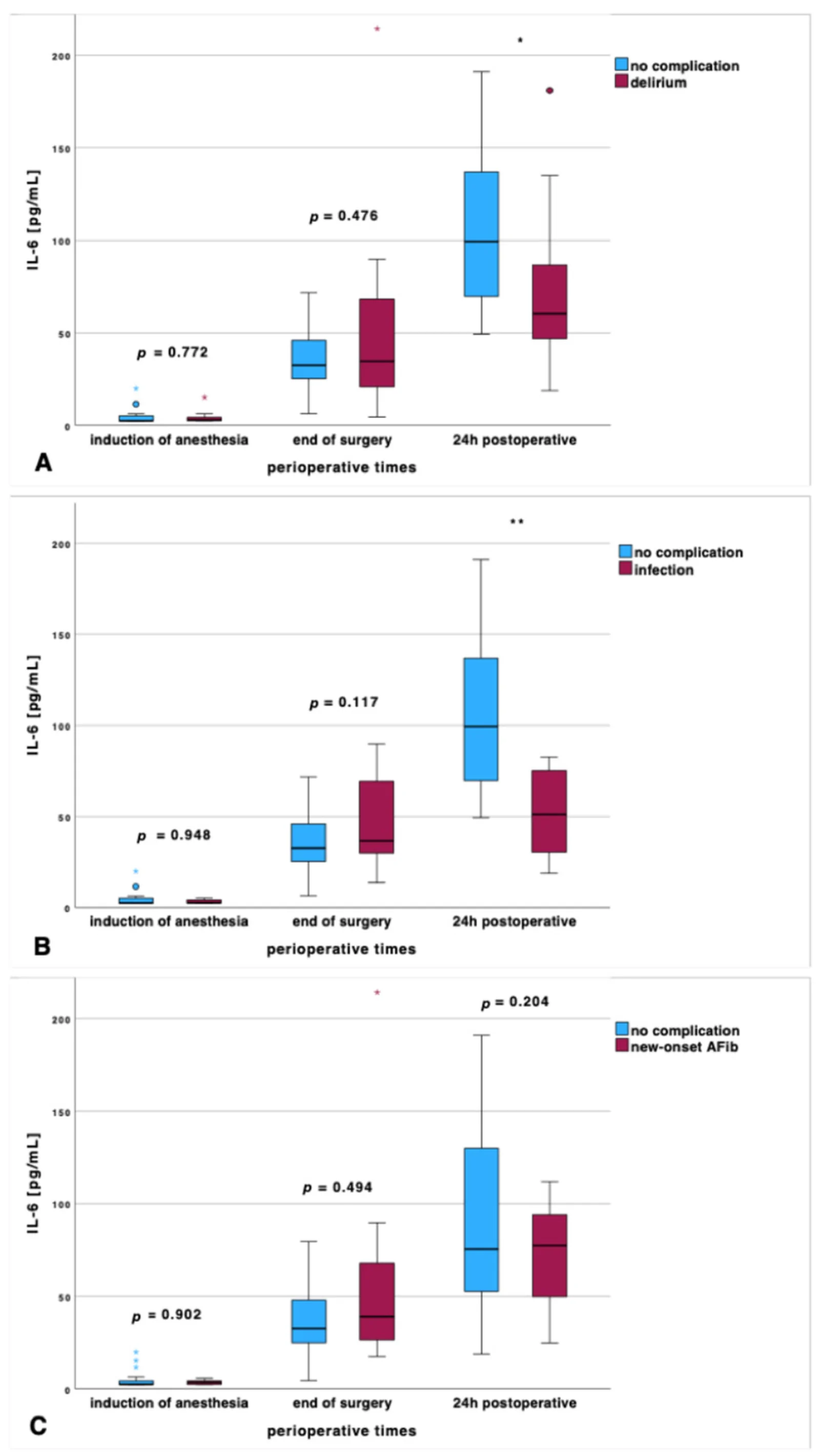
Comparison of perioperative IL-6 levels in RERACS patients without postoperative complications and RERACS patients with postoperative delirium (**A**), postoperative infection (**B**) and postoperative new-onset atrial fibrillation (**C**). *r*-value after Spearman rank correlation. (**A**): *r* = −0.2671. *p*-value after Mann–Whitney U-test. * *p* = 0.024. (**B**): *r* = −0.2572. *p*-value after Mann–Whitney U-test and *t*-test (IL-6 at the end of surgery). ** *p* = 0.001. (**C**): *r* = 0.0584. *p*-value after Mann–Whitney U-test. Abbreviations: AFib, atrial fibrillation.

Figure 1 demonstrates the comparative analysis of perioperative IL-6 levels in RERACS patients without postoperative complications (*n* = 22; 104 ± 43 pg/mL) and RERACS patients with postoperative delirium (panel A: *n* = 31; 80 ± 60 pg/mL, *p* = 0.024), postoperative infection (panel B: *n* = 17; 51 ± 23 pg/mL, *p* = 0.001), as well as postoperative new-onset AFib (panel C: *n* = 15; 105 ± 114 pg/mL). In the RERACS group, postoperative low IL-6 levels were associated with postoperative inflammatory complications such as delirium (*r* = −0.2671) or infection (*r* = −0.2572), but not with AFib (*r* = 0.0584).

Patients could contribute to more than one complication group. Among the delirium patients (*n* = 31), 38.7% (*n* = 12) also had an infection, while 22.6% (*n* = 7) of the delirium patients (*n* = 31) also had AFib. Moreover, 70.6% (*n* = 12) of patients with infection (*n* = 17) also had delirium, and 41.2% (*n* = 7) of patients with infection (*n* = 17) also had AFib. Additionally, 46.7% (*n* = 7) of the patients with AFib (*n* = 15) also had delirium, while 46.7% (*n* = 7) of the patients with AFib (*n* = 15) had infection as well. Furthermore, two patients had delirium as well as infection and AFib.

Perioperative TNF-α values in RERACS patients showed no statistically significant difference in RERACS patients with postoperative complications compared to patients without postoperative complications.

## 4. Discussion

This secondary analysis shows that ERACS with multimodal anesthesia and OTE is feasible in selected elderly patients undergoing elective cardiac surgery via (partial) sternotomy without an apparent increase in postoperative respiratory support requirement. Key findings were significantly shorter ICU-LOS, shorter duration of catecholamine dependency and high patient satisfaction, aligning with the findings of Jaquet et al. [13].

### 4.1. Clinical Outcomes

Length of stay and hemodynamic stability.

Opioids and sedatives can negatively affect hemodynamic stability [13]. Prolonged mechanical ventilation using positive-pressure ventilation may also have a negative impact on cardiac hemodynamics [13]. Hence, reducing opioids and sedatives as well as shortening mechanical ventilation time (Table 3) by promoting OTE in the RERACS program might be important factors for reduced catecholamine dependency and shorter ICU-LOS in the RERACS group compared to the control group. Jaquet et al. also reported an association between OTE, reduced vasopressor requirements and shortened ICU-LOS in patients undergoing ERACS treatment [13]. However, postoperative lower NTproBNP levels in the RERACS group reached no statistical significance (*p* = 0.452).

Improved ICU throughput streamlines surgical care for subsequent patients, reducing delays of scheduled interventions, especially for cardiac and tumor surgeries, improving institutional efficiency and elevating overall patient outcomes by enabling timely care. The RERACS concept offers alternative perioperative pathways for different cardiac surgical patient groups, especially in centers with dedicated post-anesthesia care units.

Pain and postoperative nausea and vomiting.

Opioids can cause PONV as an adverse effect [14]. Additionally, higher pain scores are associated with PONV [15]. Thus, multimodal analgesia with parasternal wound infiltration may reduce opioid requirements while achieving effective pain compensation [16]. Postoperative oxycodone requirements were equal in the two groups, and 92.3% of RERACS patients reported sufficient pain compensation in PROMs. Lowering the risk of opioid-induced adverse effects using multimodal analgesia [14] combined with administering PONV prophylaxis [17] was associated with significantly lower incidence of PONV in the RERACS group compared to the control group that received standard care without systematic PONV prophylaxis (Table 3 and Table 4). However, the definition of PONV is primarily based on the postoperative administration of antiemetic drugs. As drug administration is highly dependent on clinician practice, interpretation of the findings is limited.

Postoperative cognitive dysfunction.

To enhance postoperative neurocognitive recovery in the RERACS program, perioperative fasting and sedation times were shortened [18]. Performing OTE, early restoration of circadian rhythm and reorientation are promoted to prevent postoperative delirium. Lu et al. determined that supporting circadian rhythm along with administering dexmedetomidine [19] and low-dose ketamine [20] for intraoperative sedation is associated with improved postoperative cognitive outcomes [19]. Ketamine has neuroprotective effects [21] that promote the recovery of damaged nerves [22]. Applying low-dose S-ketamine (0.25 mg·kg^−1^·h^−1^) intraoperatively, Ju et al. observed less postoperative inflammatory cytokines, CRP and delirium [23]. Implementing multimodal RERACS treatment, we also detected significantly lower postoperative CRP levels in the RERACS group than in the control group (Table 5). As CRP is known as a delirium-related protein [24], low CRP levels may reflect a reduced or delayed systemic inflammatory response. Further studies incorporating additional measurement time points are needed to evaluate whether this finding is potentially associated with improved neurocognitive recovery. Postoperative (subsyndromal) delirium via ICDSC screening was lower in the RERACS group compared to the control group but did not reach statistical significance (Table 3). In PROMs, 34.6% of RERACS patients reported confusion and cognitive dysfunction.

However, in our current study, delirium was evaluated retrospectively. To guarantee a better understanding of postoperative delirium, future studies should include a prospective delirium screening.

Respiratory complications.

Longer mechanical ventilation correlates with more ventilation-associated pneumonia, dysphagia, longer hospital LOS and higher morbidity and mortality [25], while OTE was found to be associated with a lower incidence of postoperative pneumonia [13,26]. Diverse studies showed that preoperative inspiratory muscle training decreases the postoperative risk of pneumonia, atelectasis [27,28,29] and overall respiratory complications [29,30]. Moreover, reducing opioids and anesthetics lowers the risk of respiratory depression [31,32] and sleep-related breathing disturbances, which is especially relevant in patients with (undiagnosed) obstructive sleep apnea [31], potentially favoring postoperative respiratory stability [13].

Hence, perioperative inspiratory muscle training, intraoperative lung protective ventilation, OTE and opioid-sparing multimodal analgesia may have been important factors for reduced pulmonary complications in the RERACS group.

Unplanned re-intubation did not occur in RERACS patients. Comparing NIV prevalence and duration, no significant difference between the two groups was found (Table 3). Hence, performing OTE may be feasible in selected elderly patients without an apparent increase in postoperative respiratory support requirements compared with patients with conservative, prolonged mechanical ventilation.

### 4.2. Exploratory Inflammatory Outcomes

IL-6, TNF-α and CRP play important roles in perioperative inflammatory processes. After tissue injury, macrophages and monocytes release IL-1 and TNF-α, inducing IL-6 production, activating a systemic acute-phase response [33]. As IL-6 is rapidly released and peaks 24 h post-trauma [34], postoperative IL-6 levels may have a potential predictive association with complications, helping determine high-risk patients [33,34].

Our exploratory inflammatory analysis showed significantly lower postoperative CRP levels in the RERACS group (Table 5). Twenty-four hours after surgery, when IL-6 values usually peak [34], postoperative IL-6 levels were significantly lower in RERACS patients with postoperative inflammatory complications like delirium (Figure 1A) and infection (Figure 1B) compared to RERACS patients without complications. However, new-onset atrial fibrillation (Figure 1C) showed no significant difference in postoperative IL-6 levels compared to patients without complications.

Elderly patients (aged >65 years) can exhibit a reduced and delayed postoperative inflammatory response with lower IL-6 and other inflammatory biomarker levels [35] compared to younger individuals [33,34]. This physiological phenomenon, called immunosenescence, reflects diminished immune function, including a weakened adaptive immune response and blunted anti-infection ability [36]. CPB further disrupts pro- and anti-inflammatory immune balance, known as immunoparalysis [37,38,39], which increases susceptibility to severe systemic inflammation, e.g., sepsis, secondary infections and morbidity [40]. Therefore, in our current study, lower IL-6 levels at 24 h postoperatively, which were associated with postoperative inflammatory complications, might possibly indicate immunosenescence or immunoparalysis. Consequently, low IL-6 levels may not always be a reliable predictor for a complication-free postoperative course. Instead, the possibility of immunoparalysis or the phenomenon of immunosenescence should also be considered when interpreting inflammatory markers in elderly patients. Hence, future studies are necessary to examine our exploratory findings regarding a potential prognostic association of IL-6 with postoperative inflammatory complications in elderly patients and the immunomodulatory effects of multimodal analgesia in on-pump cardiac surgery in greater detail.

### 4.3. Patient-Reported Outcome Measures

PROMs evaluated on the second postoperative day provide insight into the course of patients’ recovery regarding their self-reported health status. Regarding pain management, patient care, preoperative patient information and PONV, the RERACS program achieved high patient satisfaction. Sore throat and hoarseness were the main postoperative discomforts in the RERACS group (Table 4). As intubation time was found to be an independent predictor for postoperative hoarseness [41], the incidence of hoarseness might have been even higher in the control group receiving standard care (not evaluated). The impact of intubation time and intraoperative transesophageal echocardiogram probes on postoperative oropharyngeal discomfort and dysphagia in elective cardiac surgery should be evaluated in further studies.

## 5. Limitations

This is a secondary analysis of 64 elderly patients (primary analysis *n* = 114) that entered our RERACS program compared to a retrospective cohort of standard care with all known limitations of such a study design. Furthermore, the present study was a single-center experience and therefore does not allow any generalization. In addition, since the studied patients were not randomized or adjusted by multivariable analysis, we cannot exclude that some of the differences are due to selection bias (e.g., team-specific extubation decision-making, individual clinician decisions regarding initiating antibiotic/antiemetic treatment). However, since both studied groups showed comparable associations in terms of pre- and intraoperative baseline characteristics, we are convinced that the differences were primarily driven by the perioperative RERACS measurements, leading to enhanced recovery.

Outcomes were restricted to the immediate postoperative period with no long-term follow-up. Since we only evaluated PROMs in the RERACS group, no comparisons with the control group can be made, and interpretation of those findings is limited. Moreover, prospective, multicentered, randomized controlled trials including PROMs in RERACS and control patients with long-term follow-up are needed to validate our findings.

## 6. Conclusions

Data support that ERACS with OTE is feasible in selected elderly patients undergoing elective cardiac surgery with (partial) sternotomy. No apparent increase in postoperative respiratory support requirements occurred compared to patients with conservative, prolonged mechanical ventilation. The RERACS program with multimodal anesthesia and OTE was associated with shorter ICU-LOS, earlier weaning from vasoactive support, and high patient satisfaction. Further studies are needed to evaluate the immunomodulatory effects of ERACS measurements and investigate the role of IL-6 levels as a marker for immunoparalysis in elderly cardiosurgical patients.

## Figures and Tables

**Table 1:** Evaluation of perioperative laboratory parameters in RERACS group and control group.

	Preoperative	End of Surgery	24 h Postoperative
RERACS	CRP	IL-6 TNF-α	CRP
	NTproBNP		NTproBNP
	IL-6		IL-6
	TNF-α		TNF-α
Control	CRP	-	CRP
	NTproBNP		NTproBNP

Abbreviations: CRP, C-reactive protein; NTproBNP, N-terminal pro-brain natriuretic peptide; IL-6, Interleukin-6; TNF-α, Tumor necrosis factor α.

**Table 2:** Patient population.

Parameter	RERACS Group (*n* = 64)	Control Group (*n* = 67)	*p*-Value	Cramer’s *V*	Cohen’s d
EuroSCORE II	1.47 ± 0.83 1.27 (0.95)	1.74 ± 1.34 1.25 (1.10)	*p* = 0.364		*d* = −0.24
ASA-Score	3.73 ± 0.45 4.00 (1.00)	3.85 ± 0.36 4.00 (0.00)	*p* = 0.101		*d* = −0.30
Male sex	92.2%/59	85.1%/57	*p* = 0.201	*V* = 0.11	
Age (years)	70.00 ± 3.33 70.00 (12.00)	71.31 ± 4.53 71.00 (8.00)	*p* = 0.056		*d* = −0.33
BMI (kg/m^2^)	27.47 ± 4.34 27.50 (6.30)	27.57 ± 3.09 27.30 (4.10)	*p* = 0.885		*d* = −0.03
Preoperative ejection fraction (%)	56.2 3 ± 8.97 59.00 (10.00)	55.79 ± 8.39 58.00 (7.00)	*p* = 0.970		*d* = 0.05
Arterial hypertonus	62.5%/40	76.1%/51	*p* = 0.091	*V* = 0.15	
Diabetes mellitus	28.1%/18	31.3%/21	*p* = 0.687	*V* = 0.04	
Heart block	10.9%/7	9.0%/6	*p* = 0.704	*V* = 0.03	
AVR Partial sternotomy	34.4%/22 50% of AVR/11	34.3%/23 43.5% of AVR/10	*p* = 0.996 *p* = 0.724	*V* = 0.00 *V* = 0.03	
CABS	65.6%/42	65.7%/44	*p* = 0.996	*V* = 0.00	
Both ITAs	9.4%/6	9.0%/6	*p* = 0.934	*V* = 0.01	
Operation time (min)	192.98 ± 34.38 188.00 (51.50)	208.28 ± 56.48 201.00 (56.00)	*p* = 0.246		*d* = −0.33
CPB time (min)	92.96 ± 23.42 92.00 (34.00)	97.86 ± 30.70 93.00 (38.00)	*p*= 0.615		*d* = −0.18
Time until OR exit (min)	20.84 ± 6.94 19.50 (7.75)	15.36 ± 5.39 15.00 (5.00)	*p* < 0.001		*d* = 0.884

Data presented as mean ± SD and median (IQR) or percentage/parameter counts (*n*). *p*-Value after Mann–Whitney U-test and *t*-test (age, BMI) or chi^2^-test. Effect size using Cohen’s d and Cramer’s V. Abbreviations: BMI, body mass index; AVR, aortic valve replacement; CABS, coronary artery bypass surgery; ITA, internal thoracic artery, CPB, cardiopulmonary bypass; OR, operating room.

**Table 3:** Parameters of postoperative course.

Parameter	RERACS Group (*n* = 64)	Control Group (*n* = 67)	*p*-Value
Postoperative LOS			
ICU-LOS (h)	44.5 ± 39.8 24.00 (26.00)	63.2 ± 48.4 46.00 (61.00)	*p* = 0.003
Hospital-LOS (days)	9.8 ± 4.4 8.01 (4.00)	11.5 ± 7.7 9.83 (7.00)	*p* = 0.155
Postoperative catecholamine dependency			
Prevalence (%)	82.5%/52 ^1^	100%/67	*p* < 0.001
Time (h)	29.51 ± 33.19 22.00 (37.89)	50.62 ± 54.79 31.33 (43.00)	*p* = 0.008
Postoperative mechanical ventilation			
Prevalence (%)	3.1%/2	100%/67	*p* < 0.001
Time (min)	16.42 ± 130.62 0.00 (0.00)	855.63 ± 783.78 588.00 (695.00)	*p* < 0.001
Postoperative non-invasive ventilation (NIV)			
NIV-mask			
Prevalence (%)	23.4%/15	19.4%/13	*p* = 0.573
Time (min)	39.83 ± 91.35 0.00 (0.00)	76.88 ± 230.16 0.00 (0.00)	*p* = 0.705
NIV-HighFlow			
Prevalence (%)	15.6%/10	22.4%/15	*p* = 0.325
Time (min)	515.75 ± 1356.56 0.00 (0.00)	864.43 ± 1785.41 0.00 (0.00)	*p* = 0.289
Postoperative complications			
New-onset AFib	23.4%/15	32.8%/22	*p* = 0.232
Postoperative infection	26.6%/17	46.3%/31	*p* = 0.019
Unplanned re-intubation	0.0%/0	1.5%/1	*p* = 1.000
Unplanned redo-operation	3.1%/2	9.0%/6	*p* = 0.097
PONV	4.8%/3 ^1^	35.8%/24	*p* < 0.001
(Subsyndromal) delirium	48.4%/31	59.7%/40	*p* = 0.196
Oxycodone requirements (mg)	20.72 ± 17.75 18.00 (17.80)	19.39 ± 13.80 18.00 (16.00)	*p* = 0.865

Data presented as mean ± SD and/or median (IQR) or percentage/parameter count (*n*). *p*-Value after Mann–Whitney U-test, chi^2^-test or Fisher’s exact test. Abbreviations: LOS, length of stay; NIV, non-invasive ventilation; AFib, atrial fibrillation; PONV, postoperative nausea and vomiting. ^1^ One patient had missing data; percentage is based on available data (*n* = 63).

**Table 4:** Patient-reported outcome measures in RERACS group. *n* = 52.

Question Topic	Applies Completely	Applies Somewhat	Applies Hardly	Does Not Apply
Preoperative patient information	100%/52	0.0%/0	0.0%/0	0.0%/0
Sore throat/hoarseness	13.5%/7	11.5%/6	5.8%/3	71.2%/37
Nausea/vomiting	3.8%/2	3.8%/2	1.9%/1	90.4%/47
Shivering	1.9%/1	1.9%/1	1.9%/1	94.2%/49
Sufficient pain therapy	92.3%/48	3.8%/2	0.0%/0	3.8%/2
Pain outside surgical site	7.7%/4	5.8%/3	1.9%/1	84.6%/44
Friendliness of staff	98.1%/51	0.0%/0	0.0%/0	1.9%/1
Patient care	100%/52	0.0%/0	0.0%/0	0.0%/0
Awareness	0.0%/0	1.9%/1	0.0%/0	98.1%/51
Delirium/confusion	13.5%/7	13.5%/7	7.7%/4	65.4%/34

Data presented as percentage/parameter counts (*n*).

**Table 5:** Pre- and postoperative CRP and NTproBNP levels.

Parameter	RERACS Group (*n* = 64)	Control Group (*n* = 67)	*p*-Value
CRP preoperative [mg/L]	9.82 ± 27.06 1.20 (3.00)	5.36 ± 10.93 1.60 (5.00)	*p* = 0.485
CRP postoperative [mg/L]	64.73 ± 53.34 42.75 (62.20)	105.87 ± 60.13 111.00 (83.90)	*p* < 0.001
NTproBNP preoperative [pg/mL]	1263.09 ± 3710.56 371.00 (747.00)	1600.06 ± 5742.80 372.00 (1034.00)	*p* = 0.930
NTproBNP postoperative [pg/mL]	2615.11 ± 1889.07 2055.00 (2396.00)	4374.00 ± 5863.91 2396.50 (4685.00)	*p* = 0.452

Data presented as mean ± SD and/or median (IQR). *p*-Value after Mann–Whitney U-test. Abbreviations: CRP, C-reactive protein; NTproBNP, N-terminal pro-brain natriuretic peptide.

**Table 6:** Diagnostic accuracy of perioperative IL-6 levels.

Complication	Perioperative Times	AUC	*p*-Value	95% CI
Postoperative delirium (*n* = 31)	Induction of anesthesia	0.472	*p* = 0.769	0.279–0.665
	End of surgery	0.432	*p* = 0.463	0.252–0.611
	24 h postoperative	0.716	*p* = 0.025	0.549–0.883
Postoperative infection (*n* = 17)	Induction of anesthesia	0.507	*p* = 0.947	0.293–0.721
	End of surgery	0.369	*p* = 0.243	0.153–0.584
	24 h postoperative	0.856	*p* = 0.002	0.715–0.996
New-onset AFib (*n* = 15)	Induction of anesthesia	0.465	*p* = 0.739	0.269–0.662
	End of surgery	0.411	*p* = 0.391	0.202–0.619
	24 h postoperative	0.504	*p* = 0.702	0.340–0.740

Area under the curve, *p*-value, 95% confidence interval after receiver operating characteristic curve analysis. Abbreviations: AUC, area under the curve; CI, confidence interval; AFib, atrial fibrillation.

## Data Availability

The datasets analyzed during the current study are available from the corresponding author on reasonable request.

## Abbreviations

The following abbreviations are used in this manuscript:

AFib	atrial fibrillation
AS	aortic stenosis
ASA-Score	physical status classification system of the American Society of Anesthesiologists
AUC	area under the curve
AV-block	atrioventricular block
AVR	aortic valve replacement
BMI	body mass index
BW	bodyweight
CABS	coronary artery bypass surgery
CAD	coronary artery disease
CI	confidence interval
COPD	chronic obstructive pulmonary disease
CPB	cardiopulmonary bypass
CRP	c-reactive protein
DE	delayed extubation
ERACS	Enhanced Recovery After Cardiac Surgery
EuroSCORE II	European System for Cardiac Operative Risk Evaluation
Hospital LOS	total postoperative length of stay in hospital
ICDSC	Intensive Care Delirium Screening Checklist
ICU	intensive care unit
ICU-LOS	length of stay in the intensive care unit
IL	interleukin
IMC	intermediate care unit
IMC-LOS	length of stay in the intermediate care unit
ITA	internal thoracic artery
LOS	length of stay
MIC	minimally invasive surgery
NIV	non-invasive ventilation
NRS	numeric rating scale
NTproBNP	N-terminal pro brain natriuretic peptide
NYHA	New York Heart Association classification for heart failure
OR	operating room
OSAS	obstructive sleep apnea syndrome
OTE	on-table extubation
PDMS	patient data management system
POD	postoperative day
PONV	postoperative nausea and vomiting
PROM	patient-reported outcome measure
RERACS	Regensburger Enhances Recovery After Cardiac Surgery
ROC	receiver operating characteristics
SD	standard deviation
TNF	tumor necrosis factor
WHO	World Health Organization
